# Translational PK/PD for the Development of Novel Antibiotics—A Drug Developer’s Perspective

**DOI:** 10.3390/antibiotics13010072

**Published:** 2024-01-11

**Authors:** Caterina Bissantz, Claudia Zampaloni, Pascale David-Pierson, Guennaelle Dieppois, Andreas Guenther, Andrej Trauner, Lotte Winther, William Stubbings

**Affiliations:** 1 Roche Pharma Research and Early Development, Pharmaceutical Sciences, Roche Innovation Center Basel, F. Hoffmann-La Roche Ltd., Grenzacherstrasse 124, 4070 Basel, Switzerland; 2 Roche Pharma Research and Early Development, Cardiovascular, Metabolism, Immunology, Infectious Diseases and Ophthalmology (CMI2O), Roche Innovation Center Basel, F. Hoffmann-La Roche Ltd., Grenzacherstrasse 124, 4070 Basel, Switzerland; 3 Product Development, F. Hoffmann-La Roche Ltd., 4070 Basel, Switzerland

**Keywords:** antibiotics, drug development, regulatory, translational PK/PD, PK/PD index, PTA, semi-mechanistic PK/PD modeling, resistance, animal model, in vitro

## Abstract

Antibiotic development traditionally involved large Phase 3 programs, preceded by Phase 2 studies. Recognizing the high unmet medical need for new antibiotics and, in some cases, challenges to conducting large clinical trials, regulators created a streamlined clinical development pathway in which a lean clinical efficacy dataset is complemented by nonclinical data as supportive evidence of efficacy. In this context, translational Pharmacokinetic/Pharmacodynamic (PK/PD) plays a key role and is a major contributor to a “robust” nonclinical package. The classical PK/PD index approach, proven successful for established classes of antibiotics, is at the core of recent antibiotic approvals and the current antibacterial PK/PD guidelines by regulators. Nevertheless, in the case of novel antibiotics with a novel Mechanism of Action (MoA), there is no prior experience with the PK/PD index approach as the basis for translating nonclinical efficacy to clinical outcome, and additional nonclinical studies and PK/PD analyses might be considered to increase confidence. In this review, we discuss the value and limitations of the classical PK/PD approach and present potential risk mitigation activities, including the introduction of a semi-mechanism-based PK/PD modeling approach. We propose a general nonclinical PK/PD package from which drug developers might choose the studies most relevant for each individual candidate in order to build up a “robust” nonclinical PK/PD understanding.

## 1. Introduction

Antibiotic drug developers operate in a challenging environment with numerous research and development (R&D) hurdles and a market that grossly undervalues innovation. Together, these challenges render the business of antibiotic development almost unviable. Despite this, significant advances in pharmacokinetic/pharmacodynamic (PK/PD) science over the past decades have improved the efficiency of R&D and provided tools to improve the success of clinical trials. This review discusses the impact of PK/PD on antibiotic R&D and offers a drug developer’s perspective on how these tools can further contribute to antibiotic development in the future.

Antibiotics are essential medicines that underpin many areas of modern medical practice but are continually under threat from antimicrobial resistance (AMR). which is a major public health concern. Globally, 1.27 million deaths in 2019 were attributed to AMR [1], and deaths are projected to rise [2]. The greater burden is in low to middle-income countries, but all parts of the world are at risk. In 2019, the US Centers for Disease Control estimated that 2.8 million antibiotic-resistant infections occurred in the United States annually, resulting in approximately 35,000 deaths [3]. Similarly, in the European Union (EU), nearly 700,000 antibiotic-resistant infections are associated with over 33,000 deaths annually [4].

A holistic approach to addressing AMR will protect our ability to treat bacterial infections in the future, and this includes the development of new antibiotics to keep pace with emerging AMR. The World Health Organization (WHO) analyzed the industry pipeline and concluded that there were relatively few clinically differentiated antibacterial agents in late-stage clinical development against critical-priority pathogens [5,6]. In particular, the WHO has designated gram-negative bacteria as one of the biggest threats and in most urgent need of new antibiotics.

Unfortunately, despite the urgent need for new antibiotics, there remains a lack of investment in the area because of discovery and development challenges, as well as its being commercially unattractive, with the market considered broken. These challenges and potential solutions to improving this situation have been discussed at length [7]. One contributing factor is the high cost of antibiotic research and development (R&D). Lowering R&D costs will not fix the problem alone, but it is an important contributing factor to help reinvigorate this space.

Despite the gloomy picture, there have been important advances in this field, including the adoption of streamlined clinical development pathways enabled by pharmacometric science. In the not-too-distant past, the traditional antibiotic development framework typically involved relatively large, paired Phase 3 studies focusing on a clinical syndrome of interest, which were often preceded by a Phase 2 study providing clinical data in support of the Phase 3 dose selection.

Since 2015, there have been several examples of more streamlined clinical development plans leading to antibiotics being approved by the U.S. Food and Drug Administration (FDA). Meropenem-Vaborbactam was approved in 2017 for the treatment of complicated urinary tract infection (cUTI) on the basis of a single Phase 3 study of 550 patients. Plazomicin was approved the following year on the basis of a 660-patient cUTI study. More recently, in 2023, sulbactam-durlobactam was approved by the FDA for the treatment of Hospital Acquired Pneumonia/Ventilator Associated Pneumonia (HAP/VAP) due to the *Acinetobacter baumannii-calcoaceticus* complex (ABC); the pivotal trial enrolled 181 patients [8].

This trend towards more streamlined clinical development reflects provisions from the regulators (FDA and European Medicines Agency (EMA)) for agents with the potential to address unmet needs. This concept of balance between the quantity of data needed for regulatory approval and the unmet medical need was nicely described in a tiered framework by Rex et al. in 2013 [9] and featured in the 2017 White Paper from the Infectious Diseases Society of America (IDSA): Developing Antimicrobial Drugs for Resistant Pathogens, Narrow-Spectrum Indications, and Unmet Needs [10].

A fundamental aspect of streamlined clinical development is increased reliance on PK/PD data, specifically integrating human pharmacokinetic data and nonclinical infection models. While these guidelines have been updated and refined [11,12], they retain a consistent core theme: that a robust PK/PD evaluation coupled with careful human safety and PK determination can be used to supplement leaner human clinical efficacy datasets in support of registration of a new antibiotic. In these cases, nonclinical PK/PD data may be used to decide on the pivotal Phase 3 dose and dosing regimen and may provide important supportive evidence for approval.

The acceptance of streamlined antibiotic development by the regulatory authorities and leading voices in the infectious diseases community has been an important step towards revitalizing the antibiotic R&D community. From an industry perspective, the high cost of conducting traditional antibiotic development diminishing returns from antibiotic commercialization had already become prohibitive and was a factor for many large pharmaceutical companies stopping or downscaling R&D for new antibiotics. 

As the unmet need has evolved, there is now a greater focus on agents for the treatment of difficult-to-treat resistance (DTR) gram-negative bacteria (e.g., Enterobacteriales, *Pseudomonas aeruginosa*, and ABC), which are becoming increasingly resistant to first-line antibiotics such as carbapenems, cephalosporins and fluoroquinolones [13]. DTR bacteria typically infect critically ill patients with serious comorbidities and are challenging to recruit into clinical trials. Streamlined development provides a potential route to bring new antibiotics for DTR pathogens to patients. This benefit goes far beyond reducing costs—the primary benefit is a clear and achievable pathway for R&D addressing high unmet needs, which would simply be infeasible through more traditional routes.

PK/PD-enabled development provides at least two streamlined pathways for developing agents to address unmet needs such as DTR gram-negative bacteria. The most common pathway relies on clinical research in a population infected with usual drug resistance (UDR) bacteria but typically excludes DTR. A recent example is meropenem-vaborbactam, a ß-lactam/ß-lactamase inhibitor (BL/BLI) combination with the potential to treat certain DTR gram-negative pathogens, Carbapenem-resistant Enterobacterales (CRE). A Phase 3 trial was conducted using piperacillin-tazobactam as the comparator for the treatment of cUTI, and it excluded carbapenem-resistant pathogens, which would not have been treatable by the comparator [14]. A small trial with CRE was also performed with 47 confirmed CRE cases enrolled, highlighting the challenges of recruiting such patients [15]. In vitro evidence using the hollow-fiber infection model [16] and in vivo using a murine thigh infection [17], both simulating humanized drug exposures, provided convincing and complementary evidence of the potential to treat CRE. These data are especially important in demonstrating the contribution of the vaborbactam component and were taken into account by the FDA, as stated in the review documents: “The evaluation of the contribution of vaborbactam to the combination relies on in vitro microbiology, animal models of infection and hollow-fiber model [18].

The other pathway involves clinical trials with the target patient population (i.e., DTR pathogens). There are only a few examples of clinical trials conducted in populations with primarily DTR pathogens, perhaps showing this is not yet a preferred route. This route is taken by necessity, e.g., for a narrow spectrum agent for which the target pathogen already exhibits high rates of resistance. Sulbactam-durlobactam (SUL-DUR) is one such example, targeting the ABC complex, which already exhibits resistance rates to carbapenems in excess of 50% in the regions where it is most prevalent. The single Phase 3 trial enrolled 181 patients in 22 months [8]. Clearly, a traditional approach consisting of 1 or 2 Phase 3 trials, each enrolling 500+ patients, would not be feasible and would delay a potentially important medicines being accessible to patients. This smaller trial led to approval in a reasonable time frame [19] and was enabled by a robust PK/PD package. The FDA review highlights how PK/PD was used to define the dosing regimen for the pivotal study and then, using PK and microbiology data from the study, to demonstrate the investigational dose provided a high (>90%) attainment rate and was used to inform dose adjustments for patients with renal impairment [20].

Based on this new framework, the development of new antibiotics depends heavily on a robust understanding of the nonclinical PK/PD properties of the investigational agent in well-validated animal models of infection and in vitro systems. This characterization of PK/PD is used as input for pivotal dosing regimen selection and to provide complementary evidence for the potential of the agent to treat DTR pathogens. However, while there are several general regulatory guidelines on translational PK/PD strategies for the development of antimicrobial drug products to date, the regulatory agencies have provided only a limited framework on what may define a “robust” supportive nonclinical PK/PD package in the context of such a lean clinical development. 

Antibiotic development pathways often reflect the unique characteristics of the investigational agent, and it is understandable that the guidance remains at a high level and does not attempt a one-size-fits-all approach. Nevertheless, it can be challenging for antibiotic developers to design a robust package/translational plan from the outset, and this is particularly complicated for novel agents for which there is no a priori knowledge of class-specific properties. While early engagement with health authorities to discuss the translational plan is essential for antibiotic developers, an assessment of the current state of the art could provide a starting point for planning. 

In this review, we will first provide an introduction to the typically applied antibacterial PK/PD approach, highlighting its strengths, and introduce the current regulatory antibacterial PK/PD guidelines. Keeping in mind the change in the regulatory landscape and its impact on the clinical development option for a leaner clinical program but increased robustness of the nonclinical package, we then critically review potential limitations of the classical PK/PD approach as outlined in the guidelines and discuss potential options to add further PK/PD understanding and confidence. We will conclude with a proposal of a general nonclinical PK/PD package from which drug developers might choose the studies most relevant for the specific context of and challenges in each individual project in order to build up an adequate “robust” nonclinical PK/PD understanding.

## 2. Translational PK/PD for Antibacterial Development in Health Authority Guidelines

### 2.1. History and Current Approach of Antibacterial PK/PD

The goal of translational PK/PD is to understand the relationship between drug exposure and drug effect based on nonclinical in vitro and/or in vivo studies. 

The concept of utilizing the PK/PD properties of antibiotics to determine dosing schedules was first introduced by Eagle in the 1950s [21,22,23,24]. He demonstrated that penicillin has time-dependent antibacterial activity, while streptomycin and bacitracin show a concentration-dependent pattern. He also found that tetracyclines exhibit a mix of these patterns. Based on these observations, Eagle suggested that continuous infusion was the best way to administer penicillin and concentration-dependent drugs should be given in regimens leading to the highest peak concentrations.

Then, several decades later, Craig rediscovered and expanded the principles of the exposure-response relationship for antibiotics and used murine infection models to show how antibiotic classes are grouped according to the PK/PD index best correlating with efficacy. This work was elegantly summarized in the article “ Pharmacokinetic/pharmacodynamic parameters: rationale for antibacterial dosing of mice and men” in which the potential application of PK/PD principles for optimization of dosing was recognized [25] and later defined [26]. These principles are still applied today.

Around the same time, similarly, pioneering work based on in vitro pharmacodynamic effects of antibiotics was performed by the group of Otto Cars, who, over a period of 20 years, developed an understanding of PK/PD behaviors using in vitro systems from the postantibiotic effect [27], sub-minimal inhibitory concentration (MIC) effects [28] to the first in vitro models to explore pharmacodynamic properties of antibiotics [29].

**Figure 1:**
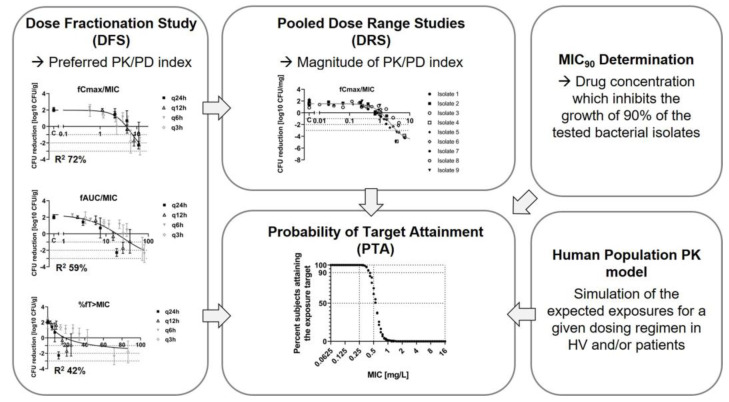
Outline of the PK/PD index approach and Probability of Target Attainment (PTA) based dose estimation integrating the nonclinical exposure target, the MIC_90_ value, and human pharmacokinetic simulations. Abbreviations: Dose fractionation Study (DFS); Dose range study (DRS); pharmacokinetic/pharmacodynamic (PK/PD); Minimum Inhibitory Concentration (MIC); free area under the curve normalized by the MIC value (fAUC/MIC); free maximal concentration normalized by the MIC value (fCmax/MIC); percentage of time during the dosing interval that the free concentration stays above the MIC value (%fT > MIC); dosing every 24 h (q24h); dosing every 12 h (q12h); dosing every 6 h (q6h); dosing every 3 h (q3h); Colony Forming Units (CFU); Probability of Target Attainment (PTA); Drug concentration, which inhibits the growth of 90% of the tested bacterial isolates (MIC_90_); Healthy Volunteer (HV).

The principles established by the groups of Craig, Cars, and other colleagues who explored its application in optimizing clinical dosing [30,31] up to breakpoint determination [32,33] remain the foundation of the PK/PD characterization of antibacterial drugs. Figure 1 provides a brief overview of this commonly used translational PK/PD approach. In the first step, dose fractionation studies (DFS) are carried out in in vivo infection models to identify the PK/PD index most strongly associated with efficacy. The three commonly used PK/PD indices for the prediction of antimicrobial efficacy are the free area under the curve normalized by the MIC value (fAUC/MIC), the free maximal concentration normalized by the MIC value (fCmax/MIC), and the percentage of time during the dosing interval that the free concentration stays above the MIC value (%fT > MIC). The MIC is determined in vitro and provides information regarding the pathogen’s susceptibility to the antibiotic. In the second part, several dose range studies (DRS) are conducted on clinically relevant pathogens to determine the magnitude of the selected PK/PD index required for efficacy (exposure target, also called pharmacodynamic target (PDT)) across a range of tested isolates. The identified exposure targets can subsequently be utilized in Probability-of-Target-Attaintment (PTA) simulations. These simulations integrate nonclinical exposure targets, MIC_90_ values based on relevant clinical isolates, and existing clinical PK data to estimate an effective dose for humans. Initially, healthy volunteer (HV) PK data might be used for the first PTA. However, since patient PK parameters might significantly differ from HVs, as soon as available, the PK properties of the specific patient populations should be used to determine an optimal dose for each population. 

The DFS and DRS studies are typically conducted in the neutropenic murine thigh and lung infection model. However, in vitro models such as the hollow-fiber infection model (HFIM) or chemostat can also be utilized, and other in vivo models might be necessary [34]. The advantage of in vitro models lies in the possibility to simulate human PK profiles, thus allowing testing the effect of the clinical concentration-time profile against isolates of interest in a nonclinical setting, and in the possibility to conduct studies over an extended treatment duration to test suppression of emergence of resistance. A detailed description of the tools used in translational PK/PD is beyond the scope of this review and has been provided in an excellent pair of minireviews by Bulitta and Rizk [35,36], which summarized findings from a workshop entitled “Pharmacokinetics-Pharmacodynamics (PK/PD) for Development of Therapeutics against Bacterial Pathogens” that was organized by the US National Institute of Allergy and Infectious Diseases. 

Best practices have been established to standardize the application of typical in vivo PK/PD models [36,37,38]. The neutropenic murine thigh and lung infection models have demonstrated their ability to predict the clinical exposure targets for multiple established antibiotic classes [39,40], and a positive correlation has been described between clinical cure, mortality, and achievement of exposure targets [41]. It stands to reason that the established translatability across multiple classes of antibiotics, from bacterial load reduction in vivo to positive outcomes in clinical trials, would also hold true for antibiotics acting through a novel Mechanism of Action (MoA). This translatability, however, has yet to be demonstrated because very few novel MoA antibiotics reach evaluation in Phase 2 or Phase 3 clinical trials. Antibiotic developers working with a novel MoA antibiotic have the benefit of these established tools at their disposal but should take this uncertainty inherent with novel classes into account and consider additional nonclinical mitigation work, especially for antibiotics being developed via a streamlined pathway for high unmet need pathogens such as *P. aeruginosa* and ABC.

### 2.2. Antibacterial PK/PD in the Regulatory Context

Two primary regulatory guidelines on translational PK/PD strategies for the development of antimicrobial drug products for Europe and the US are currently the EMA “Guideline on the use of pharmacokinetics and pharmacodynamics in the development of antimicrobial medicinal products. European Medicines Agency, EMA/CHMP/594085/2015, February 2017” [42], and the FDA “Antibacterial Therapies for Patients with an Unmet Medical Need for the Treatment of Serious Bacterial Diseases—Questions and Answers (Revision 1) Draft Guidance for Industry. FDA, CDER May 2022” [11]. There are also several other guidelines that mention the importance of translational PK/PD but do not provide details on expected strategies; these are not further discussed here.

The PK/PD index approach described above (Figure 1) is a key element of the EMA guideline. It provides some guidance for selecting the pathogens to use in the nonclinical studies, different in vitro and in vivo PK/PD models, as well as different endpoints. It does not, however, provide explicit recommendations on these topics; instead, it leaves the decision and justification to the Sponsor. Notably, the guideline includes the statement that “it is recognized that sponsors may propose alternative strategies to those outlined in this Guideline, in which case discussion with EU Competent Authorities would be appropriate”, allowing for alternative or additional approaches to the PK/PD index analysis, in case there are significant concerns about this analysis for a specific project.

The FDA guidance does not discuss PK/PD topics as thoroughly as the EMA guideline. This guidance provides a list of important data, including the evaluation of PK/PD relationships in animal models of infection, such as the PK/PD index linked with efficacy in a relevant animal model (and/or in vitro model(s)), and the target value of the PK/PD index associated with efficacy in the animal model. Similarly, to the EMA guideline, the US guidance implies that the FDA currently expects PK/PD packages to be based on the PK/PD index approach, determining the most suitable PK/PD index and its exposure magnitude (PDT) linked with efficacy. The guidance directly specifies the need to determine the exposure target in an animal model and explicitly requires studies using in vitro models of infections (e.g., HFIM) to evaluate the dose and frequency of administration using humanized PK. This is not an explicit requirement in the current EMA guideline.

Based on the strong history of the PK/PD index approach and, consequently, its implementation in regulatory guidelines, this approach is regularly employed for development candidates or marketed antibiotics (Table S1) [18,22,43,44,45,46,47,48,49,50,51,52,53,54,55,56,57,58,59,60,61,62,63,64]. It has served as a foundation for rational drug development of new antibiotics as well as to refine the dosing regimen for existing antibiotic treatments. The effectiveness of this methodology is highlighted by the observation that achieving a high PTA increases the chance of regulatory approval [65].

Recently approved antibiotics for gram-negative infections illustrate the new regulatory approach of complementing a leaner clinical development program with nonclinical PK/PD for pivotal dose selection and as additional evidence of efficacy for approval (Table S1). However, these antibiotics belong to established antibiotic classes, for which the classical PK/PD index approach has been validated by previously successful examples. In the case of the new BL/BLI combinations, specific prior nonclinical and clinical knowledge on the established BL paired with a new BLI often contributes to the PK/PD understanding of the new combination (Table S1). When developing novel antibiotics with a new MoA, there is an increased uncertainty in the adequacy of the PK/PD index approach as no prior clinical validation of the PK/PD index approach for the new MoA is available, which becomes particularly critical when relying on the nonclinical PK/PD for pivotal dose selection and as additional evidence of efficacy for approval in the context of a lean clinical development approach. In such cases, a thorough examination of potential limitations of the PK/PD index approach is warranted along with considerations of additional risk mitigation studies and/or complementary PK/PD modeling strategies.

## 3. Potential Limitations of the PK/PD Index Approach and Risk Mitigation Strategies

Several reviews discussing the limitations of the PK/PD index approach have already been published [66,67,68,69], and semi-mechanistic PK/PD modeling is often suggested as an alternative to overcome the limitations of the MIC-based PK/PD index approach. However, from a drug developer’s perspective, a complete transition from the PK/PD index approach to the semi-mechanistic PK/PD modeling may pose challenges or may not be entirely suitable. To the best of our knowledge, there are no case reports so far that demonstrate a prospective application of the semi-mechanistic approach to define the human dosing regimen or PK/PD breakpoint of a new antibiotic, nor a retrospective case report demonstrating its ability to explain suboptimal efficacy outcomes in clinical trials. In light of this lack of regulatory precedence and clinical validation, regulatory bodies and, in turn, antibiotic developers continue to rely on the commonly used MIC-based PK/PD index approach. However, regulatory bodies have recently expressed a clear interest in further advancing the mechanistic approach. As such, we propose that both approaches could be utilized in a complementary manner: If needed, specific risk mitigation activities/studies could be integrated into the regulatory accepted PK/PD index approach to address its potential limitations while in parallel, semi-mechanistic PK/PD modeling could be performed alongside the PTA- based dose selection for additional confidence.

### 3.1. Risk Mitigation by Conducting Additional Studies/Enhanced Study Design

#### 3.1.1. Incorporating Resistance in PK/PD Assessments

**Figure 2:**
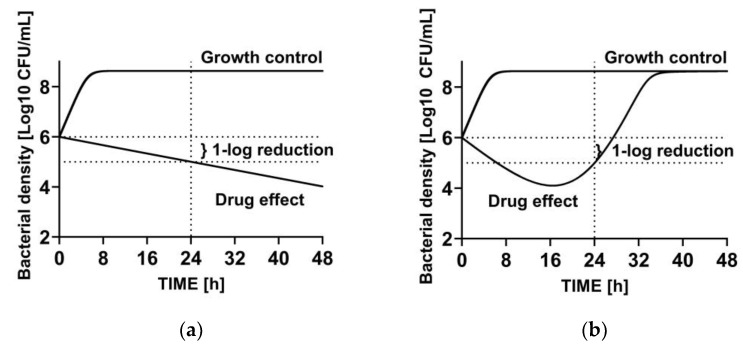
Illustration of two different bacterial killing kinetics leading to 1-log10 reduction of the bacterial density at 24 h. (**a**) Constantly progressing slow kill. (**b**) Rapid initial kill followed by regrowth.

The selection of the relevant PK/PD index and the exposure targets typically depends on the measurement of the total bacterial Colony Forming Units (CFU) after 24 h, but it does not integrate any kinetic information. This might be less critical if a complete kill would be used as a target endpoint, which is, however, unlikely to be achieved by many antibiotics. Therefore, a smaller CFU reduction, such as net stasis or 1-log10 CFU reduction, is often targeted. Due to the lack of time course information, it is then unclear whether the residual bacterial load is associated with a slow but continued bacterial killing or if the residual bacterial load is due to an outgrowing population with reduced susceptibility to the test drug (Figure 2). The latter case is highly problematic since it may describe a suboptimal treatment effect after 24 h, potentially leading to an increased risk of treatment failure in clinical trials. The emergence of resistance in even one from the panel of bacterial isolates used in PK/PD target identification can impact the overall PTA estimation [70]. However, single endpoint determinations of a stochastic process do not provide sufficient context to truly address the potential risk of resistance.

Therefore, for selected in vivo studies involving critical isolates, one should consider including additional time points to better evaluate growth and killing kinetics, especially if the exposure target is based on net stasis or 1-log10 reduction.

While the in vivo time-kill study can aid in mitigating the risk of an apparent rapid onset of regrowth, it provides neither an explanation for any observed regrowth nor eliminates the risk of regrowth at a later time of treatment. Regrowth, especially when observed in in vivo acute models, could prove terminal to a program under development and should be investigated further as it indicates either the emergence of resistance or an adaptive phenotypic response to the tested antibiotic. Antibiotic resistance is normally mediated by genotypic changes and can be detected as an elevated and often stable MIC. In contrast, transient resistance due to a phenotypic adaptation (e.g., upregulation of efflux pumps, target modulation, or derepression of resistance genes) may occur but does not always lead to an increased MIC value, especially once the drug pressure is removed [71,72,73]. This could be overlooked by MIC determination. To detect and differentiate potential mechanisms behind regrowth, we should consider complementing the determination of the total CFU count at each time point from an in vivo time-kill study with parallel CFU determination on drug-containing agar plates. The MIC of isolated colonies from both plates should be performed both after initial isolation and following drug-free passage. Importantly, as far as possible, all isolates should be analyzed by whole genome sequencing to identify the genetic basis (if any) of the observed resistance.

The distinction between resistance and adaptation is informative as it helps to identify the most effective strategy to tackle the observed re-growth. Furthermore, it provides a rationale for defining the structure of semi-mechanistic PK/PD models [74,75,76,77]. 

However, given that time course in vivo models are often acute (24–48 h) and performed with limited bacterial population sizes, they provide a useful but incomplete model to address the emergence and suppression of resistance over typical antibiotic treatment exposures and durations. In vitro models, whether static time-kill experiments or dynamic kill kinetic models using a chemostat or HFIM, provide a more thorough and versatile set of tools that can allow us to more readily assess the emergence of resistance at clinically relevant drug concentration-time exposures and durations [36]. 

Several studies have been performed to identify PK/PD-based approaches to suppress the emergence and amplification of resistant mutants [78,79,80,81]. Most of these studies were performed post-hoc and did not influence dose selection. Instead, they aimed at identifying the drivers and the magnitude of drug exposure needed to suppress the resistant populations with the ultimate goal of optimizing the recommended dosing regimen.

In general, the studies looking at the exposure required to suppress amplification of resistance using in vitro infection models indicate that PK/PD targets associated with stasis or 1 log kill at 24 hrs may be associated with the emergence of resistance. This indicates the need for higher exposures to suppress resistance amplification [80]. The magnitude of the exposures required to suppress the emergence of resistance varies depending on the antibiotic class, the species, the duration, and the test system. Of note, suppressing resistance in gram-negative organisms appears to require higher exposures than in gram-positive organisms [82]. 

Different approaches have been proposed to define exposure thresholds for suppression of resistance, such as the time over mutant preventing concentrations (T > MPC) or using the AUC/MIC ratio necessary for a 2-log kill [83,84,85,86]. However, these cutoffs may not be systematically applicable to all antibiotics due to differences in their PK/PD indices and in the way resistance develops [87,88]. The bacterial load during infection is another consideration. The high bacterial burden (>1 × 10^8^ CFU/mL) at which most of the studies demonstrate a risk of resistance amplification represents a burden that is difficult to contextualize to human infection and may not be commonly achieved in most human infections (e.g., bacteremia) [89,90] but could be attained in serious infection such as VAP [91,92].

**Figure 3:**
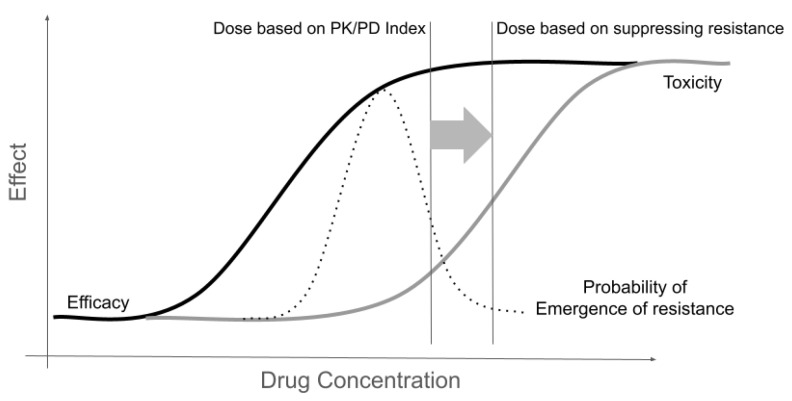
The interplay of drug concentration and effect in terms of efficacy (measured as a partial net reduction in bacterial load at a single time point), the potential of the emergence of resistance (presence of isolates with elevated MIC), and safety (measured in terms of pertinent adverse events).

In practice, the application of these concepts is not trivial, as the exposures required to achieve MPC and suppress the emergence of resistance usually exceed the doses associated with clinical efficacy, posing potential safety concerns (Figure 3). Furthermore, increasing the exposure to suppress the emergence of resistance may simply not be achievable in critically ill patients with altered pharmacodynamics or due to limited therapeutic windows [81].

Considering the increased risk of adverse events, the use of greater PK/PD targets to minimize resistance must be carefully evaluated in clinical practice and only be limited to the situation where it is beneficial: when the risk is high with the recommended dosing regimen (e.g., risk of high burden with high MIC gram-negative organisms).

Alternatively, a comprehensive understanding of the PK/PD drivers correlating with resistance suppression in conjunction with the optimal PK/PD target for efficacy might help optimize recommended dosing regimens by adjusting frequencies or infusion times [79]. An increasing number of in vitro and in vivo studies are evaluating the drivers associated with the suppression of resistance for different classes of antibiotics. It indicates that in some cases, like for aminoglycosides, the dosing regimen could be successfully optimized to minimize both resistance development and toxicity [79]. However, evidence from controlled randomized clinical trials regarding the clinical outcomes remains too sparse to validate a robust methodology [93].

Due to the scarcity of clinical validation and the lack of consensus on the resistance drivers, it is still unclear how preclinical PK/PD studies might be used to identify regimens that could minimize resistance development without compromising safety or treatment outcomes during the development of new molecules [78]. Moreover, integrating resistance suppression into the standard PK/PD approach can be complicated when indices and exposure targets between efficacy and resistance differ or do not correlate well [79,94,95].

Overall, the risk of resistance development is difficult to predict based on a single assay. However, favorable results from a variety of different studies (e.g., spontaneous mutation frequencies, induction of mutation studies, HFIM studies, MIC determination at the end of in vivo PD study) can help understand and minimize the risk of later resistance appearance at the selected dose level. It is important to note that resistant mutants appearing in in vitro systems should be phenotypically and genotypically characterized to understand the potential mechanism of resistance and evaluated in vivo in terms of their biofitness and potential for treatment by the drug. 

At present, in vitro, findings need to be narratively integrated with outcomes of existing in vivo PK/PD models to provide an informed assessment of the potential for the emergence of resistance and its suppression. We should endeavor to strengthen the integration of these data and continue developing in vivo models for the emergence of antibiotic resistance. While significant steps have already been made in this direction [96,97], there is still a need to establish the clinical validity of such studies. Such models should provide a benchmarking context within which to assess an antibiotic. If relevant, such approaches should offer a system for the reliable emergence of resistance in vivo, which could serve as a testing ground for PK/PD hypotheses to impact the emergence of resistance positively.

#### 3.1.2. Considering the Drug Concentration-Time Profile

The fundamental concept of the PK/PD index is a simplification of the drug concentration-time profile into summary PK parameters, i.e., fCmax, fAUC, %fT > MIC. However, this simplification overlooks that the drug effect might depend on the full concentration-time profile. The PK/PD index determined as most relevant in a dose fractionation study as well as its magnitude linked with efficacy might be influenced by the PK parameters and dosing frequencies applied in the DFS. Indeed, using semi-mechanistic PK/PD model-based simulations, Nielsen et al. demonstrated this using an example of six antibiotics for which the selection of the best PK/PD index and its magnitude required for efficacy is sensitive to PK differences in subpopulations and the investigated dosing interval [98]. Similarly, Kristoffersson et al. showed that the best PK/PD index of meropenem with *P. aeruginosa* depends on the dosing frequency and PK parameters, such as mice versus human parameters, with fAUC/MIC being increasingly relevant with increasing dosing frequency and increasing half-life [99]. Therefore, achieving the same exposure target value based on a murine PK or a human PK profile might not always result in the same level of efficacy. Gerber demonstrated this for different beta-lactams and aminoglycosides in a thigh infection model by administering the same dose either as a single bolus (based on murine plasma PK) or as fractionated dosing (approximating a humanized plasma PK profile) [100]. Interestingly, for beta-lactams, the humanized PK profile was superior, while for aminoglycosides, the murine plasma profile tended to achieve higher efficacy. Furthermore, Deziel et al. demonstrated with levofloxacin that different approaches to achieve human-like exposure profiles in animals (guided by the AUC(0–24 h)/MIC ratios) did not result in equivalent efficacy [101], and Felton et al. showed that the minimal free plasma concentration divided by the MIC (fCmin/MIC) ratio required to achieve stasis, 1-, 2-, and 3-log bacterial killing and suppression of emergence of resistance varied between bolus and continuous infusions of piperacillin/tazobactam against *P. aeruginosa* [102]. Conducting a meta-analysis of preclinical data in the literature, Dhaese et al. observed that intermittent and prolonged infusions of β-lactam antibiotics require different PK/PD targets to obtain the same level of bacterial cell kill [103]. These examples highlight that efficacy is influenced by the full concentration-time profile and that translating from nonclinical efficacious exposure to a clinically efficacious dose by achieving a summary PK value bears some risks. Indeed, while for beta-lactamsfT > MICfT > MIC, the nonclinical exposure target has been defined as 50–70% fT > MIC in critically ill patients, a higher exposure of 100% fT > MIC is linked with improved outcomes [104,105,106].

A potential indication of a relevant impact of the overall concentration-time profile on efficacy could be that two or more of the PK/PD indices correlate reasonably well with the efficacy readout, i.e., it is not possible to identify a unique PK/PD index without any doubt on the potential relevance of a second one. For quinolones, it is, for example, difficult to distinguish between fAUC/MIC and fCmax/MIC [107,108]. In the case of the new aminoglycoside plazomicin, based on a DFS in the neutropenic murine thigh infection model, fAUC/MIC was selected as the relevant PK/PD index for the PTA simulations [60]. While fAUC/MIC correlated with bacterial density reduction with R^2^ = 0.876, fCmax/MIC also correlated reasonably well with R^2^ = 0.783. Indeed, previous literature describes that for aminoglycosides, both fCmax/MIC and fAUC/MIC might be relevant [109]. Conducting a DFS of the cephalosporins ceftolozane against *Escherichia coli* ATCC25922 in the neutropenic murine thigh infection model during the development of ceftolozane-tazobactam showed that, as expected for a cephalosporin, %fT > MIC correlated the best (R^2^ = 0.741) [49]. However, fAUC/MIC correlated only marginally less (R^2^ = 0.732). Based on this small difference and previous cephalosporin knowledge, %fT > MIC was chosen for PTA simulations. The PK/PD index of the BLI durlobactam used in PTA simulations of sulbactam-durlobactam was selected based on results of a DFS in the HFIM evaluating the three potential indices fAUC, fCmax and %fT > 0.75 mg/L [20]. All three PK/PD indices correlated similarly well, with R^2^ ranging between 0.865–0.918. fAUC/MIC as the second best correlating PK/PD index (R^2^ = 0.88) was chosen since being more feasible to calculate in the clinical setting than the statistically better correlating index %fT > 0.75 mg/L. These examples indicate that DFS results often do not support an unambiguous preference for a single PK/PD index. In such cases, to increase confidence in the PTA, one could consider using more than 1 PK/PD index in a PTA. Nevertheless, while this might help increase robustness in the PTA, it remains a surrogate for considering the full exposure time course and does not enable an understanding of potential interactions between the two indices.

To mitigate the translational risk of using a summary PK-based exposure target, one could consider demonstrating the nonclinical efficacy of the drug concentration-time profile provided by the selected clinical regimen by conducting in vivo studies applying human PK parameters [36,110]. Such studies have, for example, been reported for meropenem-nacubactam [111,112,113], and have been integrated into recent approval packages as additional supporting evidence of efficacy, for example for ceftazidime-avibactam [114], meropenem-vaborbactam [57,115], cefiderocol [62,116], and ceftolozane/tazobactam [50]. If conducted on a large number of isolates covering a wide range of MIC values, these studies can provide supportive evidence and increase confidence in the selection of the PKPD breakpoint [116]. 

However, different patient populations can have different PK properties, and it is not feasible to carry out humanized PK-based murine PD studies simulating all relevant patient PK properties for all potential clinical dosing regimens. Therefore, one has to carefully select which regimen to simulate. Depending on the nonclinical and clinical PK profile, it might also be technically challenging to approximate the clinical concentration-time profile reasonably well in an animal model.

The fact that efficacy depends on the full concentration-time profiles highlights that the PK/PD properties of an antibiotic are impacted by the PK properties of the compound, including clearance, volume of distribution, and half-life. This is especially important in the context of PK differences between HVs and patients due to the altered pathophysiology. There might not be one dosing regimen optimally suited to treat all patient populations. Rather, a specific regimen might be required. Also, the elimination pathway is important since it influences local concentrations, such as urinary exposure. Antibiotics for cUTI should, for example, be renally excreted to achieve sufficiently high urinary exposures.

#### 3.1.3. Use of In Vivo Models Mimicking Human Disease and Treatment

As outlined in Section 2.1, the value of the acute neutropenic murine thigh and lung infection models, which are typically used for the in vivo PK/PD index analysis, has been demonstrated with many established antibiotics classes. Most clinically useful antibiotics were evaluated in these models during research and development or post-marketing and showed good translatability to clinical outcomes [39]. 

Nevertheless, there is increasing interest in more clinically relevant in vivo disease models [36,117]. One aspect is an extended infection period to study resistance suppression of the test antibiotic treatment over a longer and clinically relevant time frame. Another aspect is to better represent the natural disease progression, allowing investigation of more clinically relevant endpoints such as survival, tissue pathology, and biomarkers, in addition to measuring bacterial load in the organ of interest. Ideally, these two developments would be combined into one model that enables a disease-relevant endpoint to be monitored over a clinically relevant treatment duration. In 2020, the FDA held a workshop to discuss progress and challenges in the development and advancement of various animal models for serious infection [118]. Several alternative models were presented, which were differentiated from the standard murine models of infection. The discussion encompassed models of longer infection/treatment duration as well as more disease-relevant models in mice and higher species (rabbit, pig) with a particular focus on pneumonia indications due to the challenges they pose in the clinic. Higher species, such as rabbits and pigs, can be rendered neutropenic for several days, have the potential to mimic life-treating infections like hospital-acquired bacterial pneumonia HABP, and also establish ventilator-associated bacterial pneumonia (VABP) with intubated animals. Treatment can be provided intravenously and, importantly, with humanized exposures for a minimum of some days up to two weeks, whilst murine models are generally limited to a maximum of one day with few examples of extended infections with treatment over days [119,120]. Importantly, these models assess the evolution of the infection, the potential of bacterial eradication and resistance development during treatment, and mortality/survival while allowing several blood samples over time. Description of the validation of these larger animal species models and their potential use is a relatively recent growing topic in the literature that demonstrates the interest and efforts in this direction [121,122].

These models are not proposed as substitutes for the existing and validated murine infection models used for PK/PD assessment based on bacterial load reduction in the “classical” approach. Instead, new models could be complementary and optional when there is a need to increase the level of confidence in the selected dosing regimen prior to interventional clinical trials by mimicking the proposed human clinical dosing regimen.

#### 3.1.4. Considering the Local Free Tissue Concentration

Efficacy is driven by local free drug concentrations at the target site, which is, however, not easily measurable in nonclinical species and in humans. Free plasma concentrations are often used as an easily accessible surrogate to establish the PK/PD relationship. This can, however, be misleading and potentially result in underestimation of dose and an increased risk of clinical failure, for example, if the penetration into tissue, i.e., the tissue-plasma ratio, is lower in humans than in the nonclinical species used to determine the nonclinical free plasma-based exposure targets. This is considered one potential factor behind the apparent worse outcomes in VABP patients in the ceftobiprole Phase 3 HABP/VABP trial, despite the fact that the selected dosing regimen was supported by a positive free plasma-based PTA [34]. Target site exposures are increasingly considered in a PK/PD analysis, and the need to determine clinical PK in relevant target sites is also highlighted in regulatory guidance documents [11,42]. For pneumonia, drug exposure in the lung epithelial lining fluid (ELF) is generally considered the relevant local tissue concentration. Human ELF drug pharmacokinetics was considered in parallel to free plasma in the PTA conducted for dose justification in the recent HABP/VABP approvals of ceftolozane/tazobactam and sulbactam/durlobactam [20,50]. Ideally, a PTA conducted to support a dose for pneumonia treatment would link human ELF PK with nonclinical ELF-based exposure targets determined in a lung infection model, but alternative approaches are also applied [35]. In the case of ceftazidime-avibactam, free plasma-based exposure targets derived from different nonclinical models, including the lung infection model, were linked in PTA simulations with free plasma clinical PK, which was justified by demonstration of sufficient ELF exposure in a Phase 1 study, with slightly higher ELF penetration in humans than in mice [54]. In the case of ceftolozane/tazobactam, free plasma-based nonclinical exposure targets determined in the thigh infection model were linked in the PTA with human ELF (and free plasma) PK [50]. In the case of sulbactam-durlobactam, free plasma based exposure targets, derived from different nonclinical models, including the lung infection model, were linked in PTA simulations with clinical ELF (and free plasma) PK, which was justified by comparing the ELF penetration of the individual components (sulbactam and durlobactam) in mice and humans [20]. 

While target site exposures should be considered in the PK/PD establishment and PTA, it is important to keep in mind the uncertainty in these values. For example, ELF concentrations are generally determined by bronchoalveolar lavage (BAL) and linked with high uncertainties due to technical challenges, the risk of macrophage lysis during sample workup, uncertainties in the dilution factor between BAL fluid (BALF) and ELF, and the difficulty of obtaining multiple ELF samples from the same individual at several sampling time points [123]. In addition, human BALF studies are often carried out as single-dose studies in healthy volunteers (HV), therefore missing potential differences between HV and patients and differences in kinetics after a single dose and at steady-state. In mice, which are commonly used as PD species, ELF sampling is a terminal procedure. Therefore, across all species, only sparse data can be obtained, which renders a precise understanding of the ELF kinetics, including inter-individual variabilities, difficult. Due to the dilution of BALF, it is moreover difficult to quantify low ELF antibiotic concentrations, which might hinder capturing potential nonlinearities in the ELF penetration since antibiotic exposures from low doses are below the limit of quantification in the diluted BALF. 

In addition, based on the assumption that ELF contains only low protein levels that could contribute to drug binding and due to the lack of measured free fractions ex vivo in ELF or in vitro in simulated ELF (sELF) matrices, the observed drug concentrations in ELF have mostly been assumed to be 100% unbound and total ELF concentrations are used in the context of PK/PD discussions [124]. However, it has meanwhile been described that antibiotics can show significant binding to proteins and also lipids in ELF [125,126,127]. Based on an in vitro assay, Keemink et al. measured the percent unbound of a set of 85 antibacterial compounds in a sELF and observed that the level of binding was dependent on the physicochemical properties of the compounds. Basic compounds showed tighter binding than neutral, acidic, or zwitterionic ones due to binding to lipids [127]. Thus, observed ELF exposures should be corrected for the fraction unbound, especially for basic drugs. 

Besides the uncertainty in the observed values themselves, another level of uncertainty arises when comparing ELF exposures across different species, for example, when using murine ELF-based exposure targets as the input for a clinical ELF PK-based PTA. The BAL protocols in mice and humans are different, which might lead to a systematic bias in the data and impact the comparability between the values.

Taken together, while it is important to determine and consider local target site concentrations in determining the PK/PD relationship and efficacious dosing regimen, the uncertainties also have to be kept in mind when deciding on the specific PTA strategy. Physiological-based PK (PBPK) modeling can be helpful to further increase the understanding of the ELF kinetics and its variabilities in ELF or other local tissues. In the case of gepotidacin and zoliflodacin, PBPK models were for example designed to model compound concentrations in target compartments (i.e., saliva) [128,129]. Nevertheless, given the high uncertainties and also since local infections might spread beyond the initial infection site, an ELF (or other local tissue) based PTA alone might not be considered as sufficiently robust and should be only conducted together with free plasma-based PTA [130]. Another consideration when conducting ELF (or other local tissue) based PTA simulations could be to acknowledge the uncertainty by being less stringent in the targeted percentage of simulated patients covered.

### 3.2. Risk Mitigation by Applying an Advanced PK/PD Analysis Approach

The described risk mitigation studies can be considered to address potential limitations of the traditional PK/PD index approach. While each of these studies and measurements provides valuable information, it remains difficult to link results between studies conducted in different in vitro and in vivo model systems and to integrate all information into one quantitative PK/PD understanding. Kinetics and variability of both bacteria density and drug exposure, as well as resistance information, remain difficult to take into account in the quantitative dose selection by PTA simulation. Furthermore, for all potential different clinical drug concentration-time profiles of interest, such as different dosing regimens or different patient subpopulations with altered PK parameters, a very large humanized PK in vitro or in vivo study would be required if one wants to investigate the expected efficacy of all potential clinical regimen prior to clinical study start, which is resource- and time-wise challenging.

**Figure 4:**
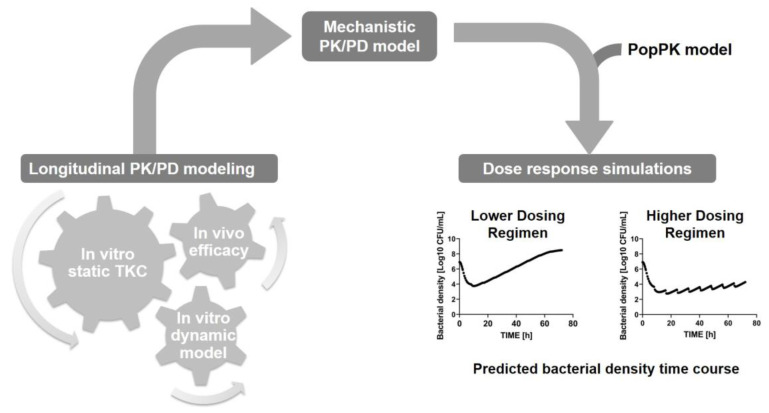
Flowchart of a general strategy to build up a semi-mechanistic PK/PD model. In vitro and in vivo PK/PD data are integrated into a PK/PD model, which allows simulations of predicted bacterial density time courses. Abbreviations: Timekill kinetics (TKC); Population PK (PopPK).

An alternative approach is the semi-mechanistic PK/PD modeling. A semi-mechanistic PK/PD model describes, by mathematical equations, the full time-course of the drug concentrations and bacterial density and allows the integration of PK/PD data from different sources into one translational PK/PD model [66,131,132]. Typically, a semi-mechanistic PK/PD model is developed by first fitting the model parameters to in vitro static time-kill kinetic (TKC) data and, if available, dynamic in vitro data before then translating to in vivo efficacy results (Figure 4). On the example of apramycin, the group of Friberg has demonstrated the strength of this approach to integrate all available efficacy data and its suitability for human efficacious dose predictions [133,134]. Mechanistic PK/PD modeling was recently listed as one of five research priorities toward precision antibiotic therapy [69].

The semi-mechanistic PK/PD approach is thus well suited to overcome potential limitations of the PK/PD index-based PTA. It avoids artificially reducing the drug exposure to the selection of one PK summary value and the resulting dependence of the efficacy from the PK parameters underlying the achievement of the PK/PD exposure targets [100]. Furthermore, it considers the expected bacterial density time course and not only CFU reduction at a single time point as the basis for dose selection. Through the consideration of bacterial time course data, it is possible to take resistance information into account to some extent. The underlying in silico model structure applied to capture potential resistance development, and its suppression is important, and it can, and should, be informed by in vitro information beyond the CFU count of the total population, such as quantification of resistant bacteria and characterization of their MIC and resistance mechanisms [135]. Considering the time course also enables the modeling of antibiotic combinations. The potentially synergistic effect of antibiotic combinations on bacterial killing and/or suppression of resistance cannot be adequately captured with traditional methods, which are based on the readout at a single time point [136,137,138].

A semi-mechanistic PK/PD model can be used to test different in silico“What-if” scenarios, such as potential clinical dosing scenarios or the impact of PK differences between healthy volunteers and patients on efficacy. Such simulations can be of importance to ensure adequate dosing regimens across all patient populations of interest.

Nevertheless, one has to keep in mind that there is no regulatory experience with this approach in the field of antibiotic development, and in vitro-in vivo translation might be challenging given the sensitivity of in vitro results to study conditions, which are not necessarily of physiological relevance [139]. Therefore, conducting both the classical PK/PD index approach as well as the semi-mechanistic PK/PD modeling in parallel might be favorable not only in terms of robustness of the PK/PD understanding of a specific development candidate but also, in general, to advance the field of antibacterial PK/PD by creating case studies allowing a real-life comparison between the two approaches.

## 4. Conclusions

The question about the “robustness” of the data package is key in the design of the nonclinical PK/PD studies and analysis, especially in the context of an antibiotic with a novel MoA and/or a lean clinical development program that relies on nonclinical data as additional supporting evidence of efficacy as a basis for regulatory approval. There is no harmonized definition of robustness, and there is no checklist for establishing such a robust package. It is rather a question for each antibiotic development candidate about the general confidence in the PK/PD understanding, efficacious exposure determination, and its translatability to clinical efficacy across all patient populations of interest.

**Figure 5:**
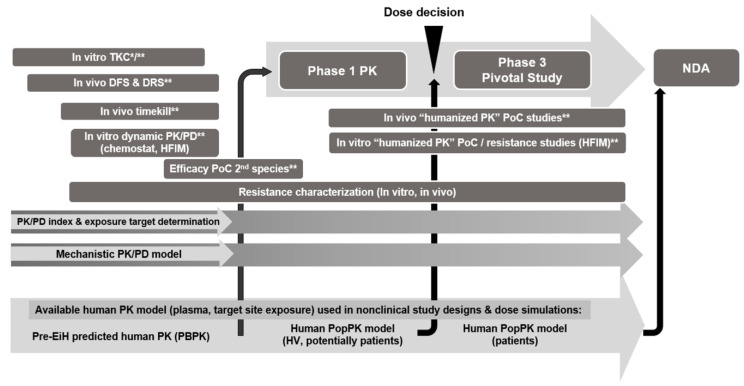
PK/PD toolbox and timing of individual studies within an antibacterial drug discovery and streamlined development project. Abbreviations: Hollow Fiber Infection Model (HFIM); Proof of Concept (PoC); Entry-into-human (EiH); Physiological-based PK (PBPK); New Drug Application (NDA); * Design to support semi-mechanistic PK/PD modeling; ** Integrate as much as possible additional readouts (for example MIC at the end of study, CFU count on drug-containing plates). Note: PK/PD analysis (index approach and semi-mechanistic PK/PD) are proposed mainly prior to EiH, but updates might arise during clinical studies; for example, based on the integration of «Humanized PoC studies», the need for including additional isolates into the PK/PD package or updated resistance information.

Based on historical experiences and current expectations of health authorities, the determination of PK/PD indices followed by exposure target definition should be part of any PK/PD package for antibacterial development. Nevertheless, this should be only the start of the PK/PD analysis. Different risk mitigation activities should be considered to address potential liabilities of the PK/PD-based dose selection, some of which are described in Section 3. Of course, not all studies are necessarily required for each development candidate; the choice should rather be based on a project-specific discussion considering the specifics of each development candidate, and these additional considerations might be especially valuable in the case of a novel MoA antibiotic following the streamlined clinical development path. Figure 5 illustrates a proposal for a “PK/PD” toolbox and the timing of individual studies within an antibacterial discovery and development project. A key objective should be to combine all available data into an integrated PK/PD understanding by means of semi-mechanism-based PK/PD modeling. This approach allows connecting results from different in vitro and in vivo models and builds up a tool that enables exploring in silico (i.e., without the need for new in vivo studies) various “What-if” scenarios. Such scenarios could include, for example, in vivo infection studies with potential clinical dosing scenarios that cannot be simulated in vivo, either due to technical limitations or due to resource constraints. 

While the focus of this review is on nonclinical activity studies, we would like to highlight that a similar in silico, in vitro, and in vivo characterization is required for safety aspects of the antibiotic to ensure that the expected efficacious exposures can be safely administered in humans.

## 5. Future Directions

The risk mitigation approaches discussed in this article are based on studies conducted in the typical in vitro and in vivo infection models currently used for PK/PD characterization. Nevertheless, we have to acknowledge that the commonly used in vitro PK/PD models (static TKC, chemostat, and HFIM) are artificial systems and do not recapitulate certain key in vivo host conditions, including anatomical barriers, the presence of an immune system, and other bacterial clearance mechanisms. This can render a direct translation of the observed in vitro activity to in vivo or clinical efficacy challenging [139,140]. For example, through omics approaches, it has been shown that the bacterial genes expressed during the human disease are not necessarily the same as observed in vitro in laboratory standard media conditions and, in some cases, in animal models due to differences in host factors. Therefore, the bacterial physiology in these models may not fully resemble human disease [141,142,143]. In this respect, enhancing the understanding of bacterial physiology during human infection is a key aspect. This area of research is important to support the establishment of new nonclinical models by understanding and optimizing translatability to treatment of infection in humans.

A major improvement of translatability could potentially become possible if we could establish bacterial infections in advanced lung in vitro models such as primary Human Airway Epithelial (HAE) cell culture models. HAE models consist of several cell types and are able to reproduce key characteristics such as anatomical barriers and mucus production. It is also possible to add immune components or other host factors to study their effects. HAE models have already been well established for antiviral efficacy by establishing viral infections in HAE models that mimic key characteristics of human infections, e.g., for treatment of RSV and coronavirus infections [144,145]. First models are meanwhile available for studying in vitro antibacterial activity in airway tissue, for example, from Epithelix [146], and warrant a thorough validation of their benefits over traditional in vitro PK/PD models. 

**Figure 6:**
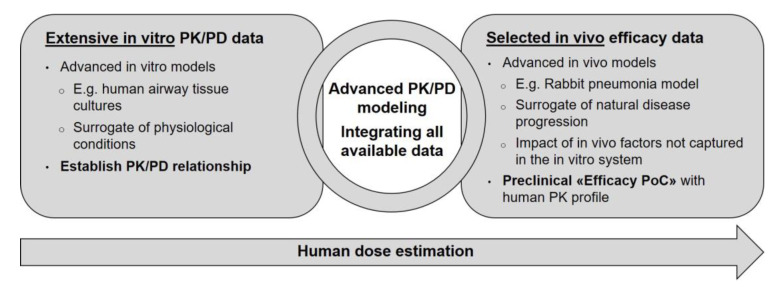
The future vision on how to design a nonclinical PK/PD package for antibacterial drug development.

Successfully establishing and validating advanced in vitro models for bacterial infections would strongly support moving away from large in vivo antibacterial PK/PD packages. Rather, PK/PD evaluations could be based on a thorough in vitro evaluation in different in vitro systems (TKC, HFIM, HAE) and be only complemented by a few key in vivo studies in advanced disease-relevant models to address questions that cannot be solved by in vitro experiments (Figure 6). Advanced semi-mechanistic PK/PD modeling could be used as a complementary method to the PK/PD index analysis to integrate all available data into one PK/PD modeling platform. 

While the focus of this review is on translational PK/PD, we do want to recognize the general important role that in silico methodologies, including Artificial Intelligence (AI), can and should play in support of modern antibacterial development. In a recent Science review, Wong et al. highlighted how Artificial Intelligence (AI) can enhance drug design and the discovery of new anti-infective agents, improve the understanding of infection biology, and accelerate the development of diagnostics [147]. In their recent viewpoint, Shuangzhe Lin & César de la Fuente presented several examples where AI and ML methods were used to design new molecules or to repurpose existing scaffolds with improved properties [148]. Similarly, in the context of antimicrobial resistance (AMR), Ali et al. and Rabaan et al. described how AI holds promise, potentially leading to faster diagnostics for resistance and more precise treatments [149,150]. Taken together, these examples demonstrate how various model-informed drug development and translational tools or approaches can support decision-making and streamline and accelerate drug development in the field of anti-infectives [151].

## Supplementary Materials

The following supporting information can be downloaded at https://www.mdpi.com/article/10.3390/antibiotics13010072/s1, Table S1: Nonclinical PK/PD models supporting dose selection and its role in recent approval packages for antibiotics developed to treat gram-negative bacterial infections.
