# Supplementary material for: Translational PK/PD for the Development of Novel Antibiotics—A Drug Developer’s Perspective
Source: Antibiotics (Basel). 2024 Jan 11;13(1):72. doi: 10.3390/antibiotics13010072 (PMC10812724; doi:10.3390/antibiotics13010072)
Supplement: Supplementary file 1 [file antibiotics-13-00072-s001.zip › antibiotics-2775717-supplementary.pdf]

Supplementary information

Translational PK/PD for the development of novel antibiotics – a drug developer's perspective

Caterina Bissantz, Claudia Zampaloni, Pascale David-Pierson, Guennaelle Dieppois, Andreas Guenther, Andrej Trauner, Lotte Winther and William Stubbings

Content:

Table S1. Nonclinical PK/PD models supporting dose selection and its role in recent approval packages for antibiotics developed to treat gram-negative bacterial infections.

Table S1. Nonclinical PK/PD models supporting dose selection and its role in recent approval packages for antibiotics developed to treat gram-negative bacterial infections.

Antibiotic	Indication (year of FDA approval)	• P2 / P3 clinical studies<br>• Role of PK/PD in the development and approval<br>• PK/PD approach for dose justification
Ceftolozane/Tazobactam[43–50]	cIAI, cUTI (2014)	• 1 P2 cUTI and 1 P2 cIAI (1 dose level in both trials only); 1 P3 cUTI and 1 P3 cIAI (following the new cIAI draft guidance 2012 and agreed by the FDA, the originally planned 2 P3 per indication were pooled into 1 P3 each)<br>• Only the P2 cIAI allowed exposure-response analysis, and a flat (no) relationship was observed. Therefore, nonclinical PK/PD played a vital role in dose justification and breakpoint determination.<br>• PK/PD: PTA based on nonclinical exposure targets derived in the neutropenic murine thigh infection model and HFIM
	HABP/VABP (2019)	• 1 P3 trial HABP/VABP with a higher dose than approved for cUTI and cIAI based on PTA simulations integrating nonclinical exposure targets and clinical ELF PK.<br>• One single trial provided substantial evidence of effectiveness since Ceftolozane/tazobactam already approved for cIAI and cUTI. Additional supportive information provided by *in vitro* and animal models of infection.<br>• PK/PD: Previously established exposure targets (neutropenic murine thigh infection model), applied in PTA to free plasma and total ELF clinical PK. The HABP/VABP dosing regimen was increased 2-fold compared to cIAI/cUTI since the ELF penetration in ventilated patients was ~50% and 63% for ceftolozane and tazobactam.<br>o Additional nonclinical studies were conducted: 1) studies in the neutropenic murine lung infection model approximating ELF concentrations of ceftolozane-tazobactam in patients, and 2) HFIM studies simulating the human ELF exposures (in 1 study ELF exposures of critically ill pneumonia patients were simulated). Together with the PTA, these nonclinical studies contributed to the breakpoint determination.
Ceftazidime/Avibactam[51–54]	cIAI, cUTI (2015)	• 2 P2 studies (1 cUTI, 1 cIAI) and 4 P3 clinical studies (2 cUTI, 2 cIAI) were conducted for cUTI and cIAI. The original FDA approval in 2015 was based on the Phase 2 data prior availability of the P3 results.<br>• No exposure-response relationship could be established based on the P2 studies, and dose selection was thus supported by nonclinical PK/PD, i.e. the PTA based on nonclinical exposure targets and clinical PK. The P3 trials then confirmed the efficacy of the selected dosing regimen.<br>• PK/PD: PTA based on ceftazidime nonclinical exposure targeted reported in the literature and avibactam nonclinical exposure targets determined in the neutropenic murine thigh and lung infection models.
	HABP/VABP (2018)	• No P2 and a single P3 study for HABP/VABP<br>• As for cUTI and cIAI, P3 dose selection was based on the PTA approach and the dose then afterwards validated by the P3 efficacy results.<br>• PK/PD: PTA-based dose confirmation using simulated clinical PK (free plasma) in HABP/VABP patients and the same nonclinical (free plasma) exposure targets as for cUTI and cIAI. Background of using plasma based exposure targets was demonstration of sufficient ELF exposure in a P1 study, with slightly higher ELF penetration in humans than in mice.
Meropenem/Vaborbactam [18,55–57]	cUTI (2017)	• No P2 study and 1 P3 (cUTI)<br>• A single P3 trial was considered adequate for several reasons including the established efficacy of meropenem in the treatment of cUTI, and data from *in vitro* studies and animal models of

		infection demonstrating the activity of meropenem-vaborbactam against bacteria relevant to cUTI.<br>• PK/PD: PTA based on meropenem nonclinical exposure targets determined in murine neutropenic infection models (including literature data), vaborbactam nonclinical exposure targets determined in neutropenic thigh infection model and HFIM (both models using humanized dosage regimen, HFIM used only for index selection but not for exposure target)<br>o Several *in vitro* (HFIM) and *in vivo* (neutropenic murine thigh and lung infection model, murine model of pyelonephritis) studies against MDR / CR isolates simulating the approved human dosing regimen were conducted during the development.
Plazomicin [58–60]	cUTI (2018)	• 1 dose-ranging P2 trial and 1 P3 trial<br>• No exposure-response relationship could be determined, highlighting the need for nonclinical PK/PD understanding.<br>• PK/PD: PTA based on nonclinical exposure targets determined in the murine neutropenic thigh infection model
Cefiderocol [61–63]	cUTI in patients with limited or no alternative treatment options (2019)	• 1 P2 (cUTI) and 1 cUTI cohort in a P3 in patients with CR gram-negative bacteria.<br>• A single adequate and well controlled trial was considered sufficient due to the additional *in vitro* and animal models of infection.<br>• PK/PD: PTA based on nonclinical exposure targets determined in the murine neutropenic thigh infection model<br>o Notably, a humanized PK study was conducted in the neutropenic murine thigh infection model and an immunocompetent rat lung infection model.
	HABP/VABP in patients with limited or no alternative treatment options (2020)	• 1 P3 (HABP/VABP)<br>• PTA based on free plasma ELF confirmed the same dosing regimen as approved for cUTI
Imipenem-cilastatin/Relebactam (IMI/REL) [64]	cUTI and cIAI in patients with limited or no treatment options (2019)	• 1 cUTI and 1 cIAI as well as one very small study with IMI nonsusceptible organisms<br>• cUTI and cIAI trial efficacy conclusions were limited since using IMI alone as comparator and the trials mainly included IMI-susceptible pathogens. Results from the IMI-non susceptible isolates trials also difficult to interpret. Nevertheless, IMI/REL was approved for patients with limited or no treatment options due to substantial evidence of effectiveness relying reliant in part previous IMI efficacy data, *in vitro* and animal data demonstrating that REL restores activity of IMI against IMI-nonsusceptible Gram-negative organisms expressing some Class A and some Class C .-lactamases, and PTA analyses for REL conducted by the FDA.<br>• PK/PD: PTA based on relebactam nonclinical exposure targets derived from the neutropenic murine thigh infection model. No exposure targets and PTA for IMI since previously approved dose and assumed that IMI PKPD remains the same if REL inhibits all beta lactamases of IMI-non susceptible isolates (FDA reviewer)
	HABP/VABP (2020)	(no review available on FDA webpage at time of writing, November 2023)
Sulbactam/Durlobactam [20]	HAP/VAP due to CR-ABC (2023)	• 1 P3 trial in HAP/VAP caused by CR-ABC with limited sample size (177 evaluable patients).<br>• FDA stated that it therefore puts greater reliance on nonclinical studies (*in vitro* and animal studies) and on the known safety profile of sulbactam.<br>• PK/PD: PTA based on nonclinical exposure targets (sulbactam PK/PD index determined in HFIM and durlobactam PK/PD index in chemostat; exposure targets determined in DRS in neutropenic murine thigh and lung infection models) and clinical free plasma & total ELF PK. Both free plasma and total ELF human PK was related to free plasma based nonclinical exposure targets, which was justified by comparing ELF/free plasma ratios in mice and human for both compounds.

Abbreviations: Complicated urinary tract infection (cUTI); complicated intraabdominal infection (cIAI); hospital-acquired bacterial pneumonia/ventilator-associated bacterial pneumonia (HABP/VABP); carbapenem-resistant (CR); *Acinetobacter baumannii-calcoaceticus*- complex

(ABC); Multi drug resistant (MDR); Hollow Fiber Infection Model (HFIM); P2 (Phase 2); P3 (Phase 3); Epithelial lining fluid (ELF); U.S. Food and Drug Administration (FDA).
